# Probiotic and anti-inflammatory potential of *Lactobacillus rhamnosus* 4B15 and *Lactobacillus gasseri* 4M13 isolated from infant feces

**DOI:** 10.1371/journal.pone.0192021

**Published:** 2018-02-14

**Authors:** Nam Su Oh, Jae Yeon Joung, Ji Young Lee, Younghoon Kim

**Affiliations:** 1 R & D Center, Seoul Dairy Cooperative, Ansan, Kyunggi, South Korea; 2 Department of Biotechnology, College of Life Sciences and Biotechnology, Korea University, Seoul, South Korea; 3 Department of Animal Science and Institute of Milk Genomics, Chonbuk National University, Jeonju, South Korea; Universite Clermont Auvergne, FRANCE

## Abstract

A total of 22 *Lactobacillus* strains, which were isolated from infant feces were evaluated for their probiotic potential along with resistance to low pH and bile salts. Eight isolates (*L*. *reuteri* 3M02 and 3M03, *L*. *gasseri* 4M13, 4R22, 5R01, 5R02, and 5R13, and *L*. *rhamnosus* 4B15) with high tolerance to acid and bile salts, and ability to adhere to the intestine were screened from 22 strains. Further, functional properties of 8 *Lactobacillus* strains, such as anti-oxidation, inhibition of α-glucosidase activity, cholesterol-lowering, and anti-inflammation were evaluated. The properties were strain-specific. Particularly, two strains of *L*. *rhamnosus*, 4B15 (4B15) and *L*. *gasseri* 4M13 (4M13) showed considerably higher anti-oxidation, inhibition of α-glucosidase activity, and cholesterol-lowering, and greater inhibition of nitric oxide production than other strains. Moreover, the two selected strains substantially inhibited the release of inflammatory mediators such as TNF-α, IL-6, IL-1β, and IL-10 stimulated the treatment of RAW 264.7 macrophages with LPS. In addition, whole genome sequencing and comparative genomic analysis of 4B15 and 4M13 indicated them as novel genomic strains. These results suggested that 4B15 and 4M13 showed the highest probiotic potential and have an impact on immune health by modulating pro-inflammatory cytokines.

## Introduction

Lactic acid bacteria, especially the species belonging to the genus *Lactobacillus*, such as *L*. *acidophilus*, *L*. *rhamnosus*, *L*. *gasseri*, *L*. *fermentum*, and *L*. *plantarum* act as important probiotic because of their strain-specific properties that are beneficial to health [1]. To function as probiotics, bacterial strains should meet certain requirements including resistance to high acid and bile concentrations [2]. Other functional properties for characterizing probiotics are bacterial adherence to intestinal epithelial cells, the production of antimicrobial compounds, and the ability to modulate immune responses [2,3,4] for instance. Probiotic strains should be able to survive in the gastrointestinal tract in sufficient numbers, and have metabolic activities that are beneficial to the host [5,6]. Previously, *L*. *gasseri* has been reported to produce a number of bacteriocins, with the most well-characterized being gassericin A from *L*. *gasseri* LA39, which was isolated from infant feces [7]. Verdenelli et al [8] isolated *L*. *rhamnosus* IMC 501 from feces of elderly Italians, and the strain showed high adhesive ability and inhibitory activities against pathogens, particularly *Candida albicans*. *L*. *casei* Zhang, which was isolated from koumiss, was also a potential probiotic with high acid resistance, bile salt resistance, gastrointestinal persistence, and cholesterol-reducing and antimicrobial activities [9]. *Lactobacillus* strains are found naturally in the human intestine, and for this reason, such strains are preferentially developed for commercial use as probiotics. Some researchers reported that bacteria isolated especially from the feces of infants or elderly humans possess potential probiotic properties [8,10]. Currently, sufficient numbers of well-characterized probiotic strains are available for commercial use around the world [11,12]. Recently, probiotics have emerged as potential, novel, and natural therapeutic drugs [4]. Thus, the isolation and characterization of new strains are still needed.

In this study, we isolated 22 *Lactobacillus* strains from infant feces, and evaluated their probiotic potential along with resistance to high acid and bile concentrations; further, the various functional properties of the selected isolates, such as adhesion to the intestine, anti-oxidation, inhibition of α-glucosidase activity, cholesterol lowering, and anti-inflammation were investigated. Additionally, whole-genome sequencing and comparative genomic analysis of the selected probiotic strains were carried out to present complete genome sequence and genetic properties.

## Materials and methods

### Isolation and identification of the *Lactobacillus* strains

A total of five healthy, exclusively new-born infants, aged under 2 weeks, were selected for the study. The present study was conducted according to the guidelines laid down in the bioethics and safety act of ministry of health and welfare (South Korea) and written informed consent was obtained from the parents after a careful explanation of the research. The fecal samples from breast-fed babies (under recruitment of the volunteers and rewritten informed parental consent) were obtained directly from diapers. This study was approved by institutional review board of Samsung Medical Center (IRB No. 2017-08-040). The samples (10 g) were weighed aseptically, and homogenized for 2 min in a stomacher (Stomacher 80 Biomaster, Seward) containing 90 ml of peptone water. Briefly, the homogenized samples were serially diluted with 0.85% NaCl, and spread or streaked on the surface of MRS (de Man, Rogosa and Sharpe) agar plates (Difco, Detroit, MI, USA), and the plates were incubated at 37°C for 48 h. A total of 8 *Lactobacillus* strains were isolated, and each strain was identified using MALDI-TOF mass spectrometry to the species level (S1 Table). The pure cultures of the isolates were preserved in MRS broth containing 50% (v/v) glycerol as a cryoprotectant at 80°C. The cultures were subcultured thrice in MRS broth prior to use.

### Determination of the probiotic properties in the gastrointestinal tract model

#### Determination of resistance to acid and bile salts

The tolerance of the *Lactobacillus* strains to acid and bile salts was tested as described previously by Zielinska et al. [2] with slight modifications. *Lactobacillus* strains were cultured for 18 h in MRS medium, and then, 1% of the cultures were transferred into 50 mM PBS (pH 3.0) with 1 N HCl, and 50 mM PBS (pH 7.0) supplemented with 1% oxgall for testing the resistance to acid and bile salts, respectively. Resistance was assessed in terms of viable colony counts, and viable colonies were enumerated using the pour-plating method on MRS agar after incubation at 37°C for 2 h with PBS (pH 3.0) and for 6 h with oxgall medium.

#### Ability of adhesion to the intestine in HT-29 intestinal cells

The ability of *Lactobacillus* strains to adhere to the intestine was determined using HT-29 cells by using a method that was described by Kim et al. [13]. The adhesion ability was assessed in terms of viable colony counts, and the number of viable cells of the *Lactobacillus* strains was counted by using the spread plate method on MRS agar.

### *In vitro* study of the functional properties of the *Lactobacillus* strains

#### Determination of the antioxidant activity

The antioxidant activities of *Lactobacillus* strains were determined by estimating the reducing power and radical-scavenging ability using the ferric-reducing antioxidant power (FRAP) assay, the 2,2-diphenyl-1-picrylhydrazyl (DPPH) assay, the 2,2’-azino-bis(3-ethylbenzothiazoline-6-sulfonic acid) (ABTS) assay, and the oxygen radical absorbance capacity (ORAC) assay. The FRAP, DPPH, and ABTS assays were performed by following the method described by Oh et al. [14], and the ORAC assay was conducted using the procedures described by Roy et al. [15].

#### Determination of the inhibition of α-glucosidase activity

The estimation of the inhibition of α-glucosidase activity was carried out as described in a previous report [16] with slight modifications. The supernatant, which was obtained through the sonication and centrifugation of rat intestinal acetone powder, was used as the source of crude intestinal α-glucosidase. The crude enzyme was mixed and incubated with the samples and the substrate (ρ-nitrophenyl-α-D-glucopyranoside) at 37 °C for 30 min. The reaction mixture was further incubated for another 5 min at 100 °C in order to inactivate the enzyme and the release of nitrophenol was read spectrophotometrically at 405 nm. Then, the inhibition of α-glucosidase activity was calculated as (1- A/B) × 100, where A was the absorbance of the reactants with test samples, and B was the absorbance of the reactants without the samples. Acarbose was used as the standard reference.

#### Determination of the cholesterol-reducing activity

A quantitative assay for assessing the cholesterol-reducing activity was conducted as described by Lee et al. [17] with minor modifications. The amount of residual cholesterol in the cholesterol medium was measured using the Total Cholesterol Assay Kit (Cell Biolabs, Inc., San Diego, CA, USA). The cholesterol in the non-inoculated sterile broth was also analyzed in order to serve as a negative control.

#### Determination of the anti-inflammatory activity in RAW 264.7 macrophage cells

RAW 264.7 macrophage cells were purchased from ATCC, and cultured in Dulbecco’s modified Eagle’s medium (DMEM) (GE healthcare Life Sciences, Logan, UT, USA), supplemented with 10% fetal bovine serum (FBS) and 1% penicillin/streptomycin at 37°C in a humidified atmosphere containing 5% CO_2_. To determine the anti-inflammatory activity, the macrophage cells (1 × 10^6^ cells/mL) were treated with 10^6^ CFU/mL to 10^8^ CFU/mL of selected *Lactobacillus* strains in DMEM medium without antibiotics for 12 h before exposure to 100 ng/mL LPS for 18 h. The bacterial samples were dissolved in the medium lacking antibiotics, and added directly to the cell culture medium. Each treatment was performed in six replicates in a single experiment. The production of nitric oxide (NO) was measured using the Griess reaction [18]. Evaluation of the concentration of the cytokines released (TNF-α, IL-1β, IL-6, and IL-10) in the supernatants of the RAW 264.7 cell culture was carried out with enzyme-linked immunosorbent assay kits (ELISA MAX Deluxe Set) (Biolegend Inc., San Diego, CA, USA) according to the manufacturer’s instructions. Additionally, mRNA expressions of above four genes were determined with quantitative real-time PCR by following the method described by Lee et al. [19].

### Whole genome sequencing

Genomic DNA extraction and whole genome sequencing of *L*. *rhamnosus* 4B15 and *L*. *gasseri* 4M13 were performed as the method described by Oh et al. [20] with slight modifications. The complete genomes of *L*. *rhamnosus* 4B15 and *L*. *gasseri* 4M13 were constructed *de novo* using Pacific Biosciences (PacBio)_20K sequencing data and the sequencing analysis was performed in Chunlab, Inc. (Seoul, Korea). The resulting contigs were scaffolded using PacBio SMRT Analysis version 2.3.0 (Pacific Biosciences, Menlo Park, CA, USA). The raw PacBio sequencing data of *L*. *rhamnosus* 4B15 and *L*. *gasseri* 4M13 were deposited at NCBI with accession numbers CP021426 and CP021427, respectively.

### Comparative genomics

A total of four reference strains for each of the selected *Lactobacillus* strains 4B15 and 4M13 were obtained from the EzGenome database (http://ezgenome.ezbiocloud.net) as the closest neighbors based on the average nucleotide identity (ANI) values, and these genomes were used for comparative genomic analysis. The phylogenetic tree was constructed based on the OrthoANI algorithm in order to access overall similarity of genome sequences of the selected *Lactobacillus* strains and the other four strains using CLgenomics^™^ software (ChunLab Inc. Korea). The dendrogram and Venn diagram were constructed using CLgenomics^™^ software based on the gene content (presence or absence) and pan-genome orthologous groups (POGs), respectively [21].

### Statistical analysis

All data are expressed as means ± standard deviation (S.D.). The statistical significance of the differences among the groups was assessed using an independent sample t-test or Duncan’s multiple-range tests. SPSS software (version 22.0, IBM, Chicago, IL, USA) was used to perform all statistical tests.

## Results

### Probiotic properties of the *Lactobacillus* strains in the gastrointestinal tract model

Probiotics are required to survive the gastrointestinal (GI) tract and colonize the intestine in order to confer health benefits. The first step in determining the probiotic properties of the *Lactobacillus* strains was the estimation of the resistance to conditions of low pH and high concentration of bile salts. 8 among total 101 isolates of *Lactobacillus* showed high tolerance to a pH of 3.0 with their survival rate exceeding 97% (Table 1). At 1% concentration of bile salts, 8 isolates were capable of growth with their survival rate ranging from 97.5% to 103.4% after incubation for 6 h. The 8 isolates were tested for their ability to adhere to HT-29 cells. All of the tested strains except 5R01 showed significantly higher adhesive abilities than the reference strain *L*. *rhamnosus* GG (65.7%) with adhesive ability exceeding 67.3%. Additionally, the assessment of the safety of the isolates was performed using urease and gelatinase tests, and urease negativity and gelatinase negativity were observed in all strains (S2 Table).

**Table 1:** Acid and bile tolerance and intestinal adhesion activity of selected *Lactobacillus* strains.

		3M02	3M03	4M13	4R22	5R01	5R02	5R13	4B15
**Acid tolerance**	0h (Log CFU/mL)	7.08	7.32	6.62	6.17	6.82	6.63	6.64	7.12
	2h (Log CFU/mL)	6.88	7.29	6.44	6.22	6.72	6.56	6.64	7.18
	Survival rate (%)	97.2	99.5	97.2	100.9	98.5	98.9	100.0	100.8
**Bile tolerance**	0h (Log CFU/mL)	7.08	7.32	6.62	6.17	6.82	6.63	6.64	7.12
	6h (Log CFU/mL)	6.98	7.14	6.45	6.18	7.05	6.65	6.79	7.08
	Survival rate (%)	98.6^de^	97.6^ef^	97.5^f^	100.3^c^	103.4^a^	100.3^c^	102.3^b^	99.4^cd^
**Intestinal adhesion activity**	0h (Log CFU/mL)	8.29	8.40	8.58	8.91	8.27	8.06	8.94	8.89
	2h (Log CFU/mL)	6.44	6.37	6.55	7.07	5.57	6.18	6.92	7.05
	Adhesion activity (%)	77.7^ab^	75.8^b^	76.4^ab^	79.4^a^	67.3^c^	76.8^ab^	77.4^ab^	79.3^a^

Data followed by a different lower case letter within a column were significantly different (P < 0.05)

### Anti-oxidation, inhibition of α-glucosidase activity, and cholesterol-lowering by the *Lactobacillus* strains

**Fig 1:**
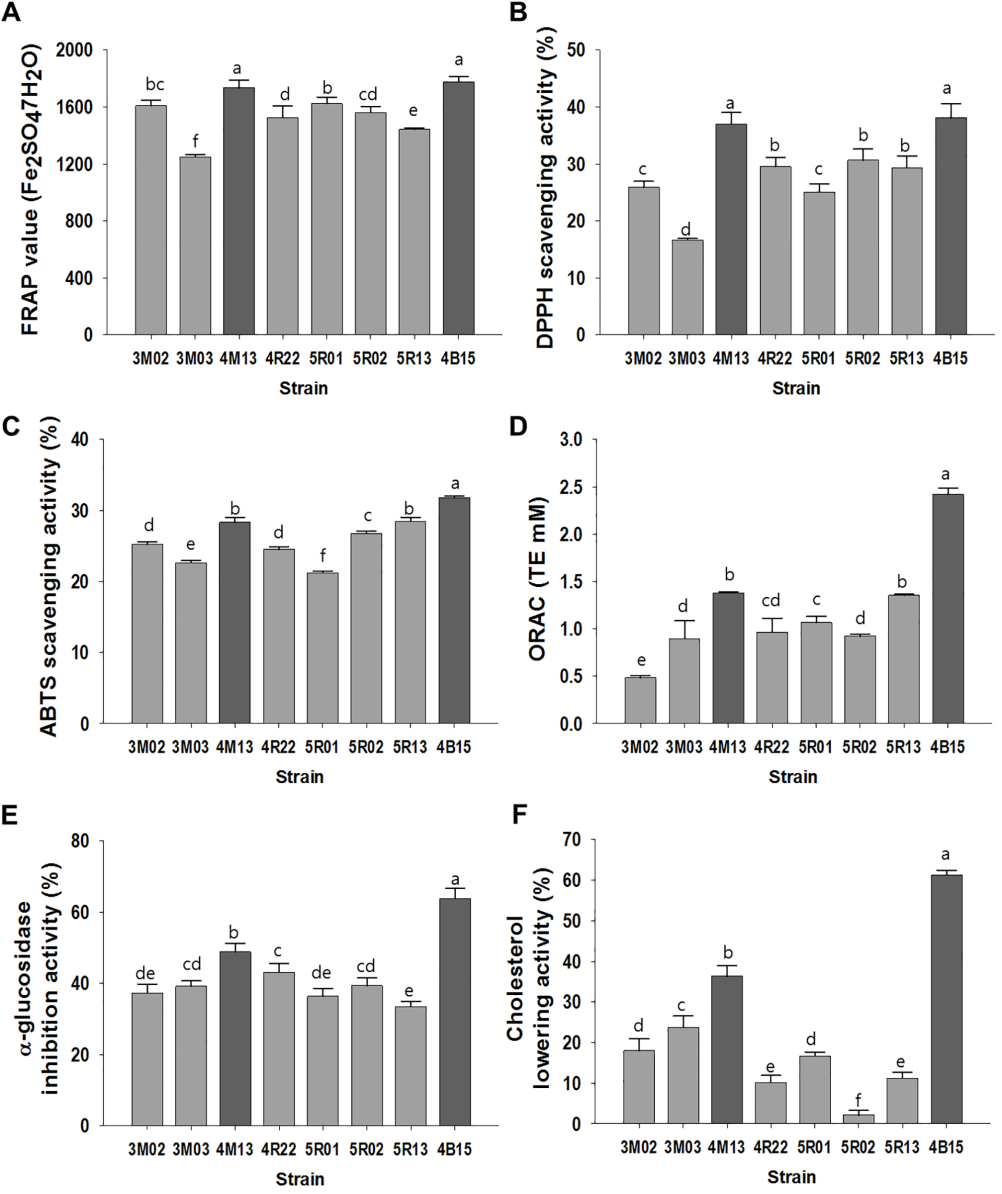
Antioxidant, α-glucosidase inhibitory, and cholesterol lowering activities of the *Lactobacillus* strains. A. FRAP assay, B. DPPH scavenging activity, C. ABTS scavenging activity, D. ORAC assay, E. α-glucosidase inhibitory activity, and F. cholesterol lowering activity. Data are expressed as mean ± S.D. from three independent experiments. Bars with different letters indicate significant differences at *P* < 0.05.

The antioxidant capacity of the isolates is shown in Fig 1A–1D, which was determined by measuring their reducing power and radical-scavenging activities. Specifically, two strains, namely, 4M13 and 4B15 exhibited considerably higher activities than the other *Lactobacillus* strains in the FRAP and DPPH assays, with values exceeding 1,734.3 μM and 37.0% respectively. Following 4B15 and 4M13, 5R13 showed high antioxidant activity in the ABTS and ORAC assays. Particularly, 4B15 demonstrated the highest antioxidant activity in all assays, with the markedly high value of 2.4 mM in the ORAC assay. Moreover, the eight isolates were able to inhibit the activity of α-glucosidase and reduce cholesterol concentration as shown in Fig 1E and 1F, with the values exceeding 48.9% and 36.3%, respectively. The activities of 4B15 were substantially higher than those of 4M13.

### Anti-inflammatory activities of the *Lactobacillus* strains

**Fig 2:**
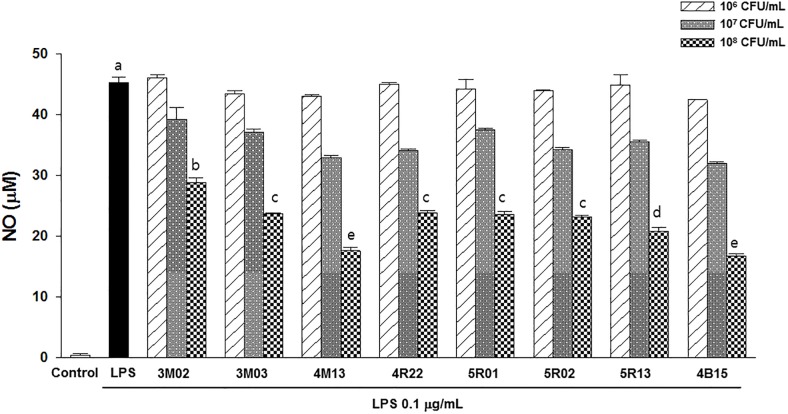
Production of nitric oxide in LPS-induced RAW 264.7 treated with the selected *Lactobacillus* strains. Data are expressed as mean ± S.D. from three independent experiments. Bars with different letters indicate significant differences at *P* < 0.05.

**Fig 3:**
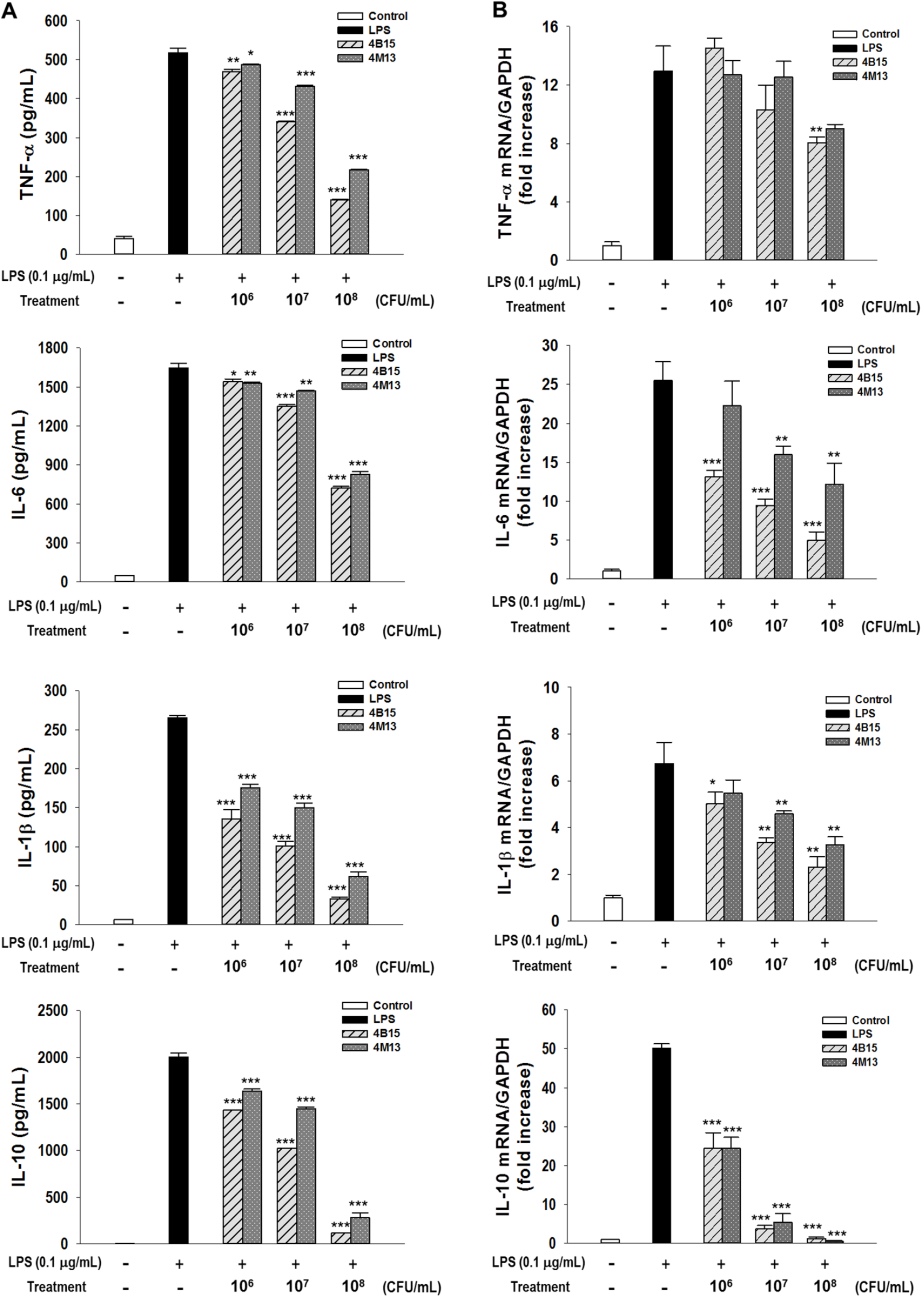
Anti-inflammatory activities of the selected *Lactobacillus* strains. A. pro-inflammatory cytokine release in RAW 264.7 cells using ELIZA, B. mRNA expression of imflammation related genes using qRT-PCR. Data are expressed as mean ± S.D. from three independent experiments. Significant difference was marked with an asterisk (*P < 0.05, **P < 0.005, ***P < 0.001).

To determine the anti-inflammatory activities using RAW 264.7 macrophages, the cytotoxicity of the *Lactobacillus* strains was evaluated, and no effect on cell viability was observed at the concentrations of the bacterial isolates used in the study, as determined by the MTT assay (S1 Fig). Therefore, the production of NO and pro-inflammatory cytokines and the expression of mRNA in the LPS-stimulated cells at the three concentrations of isolates was investigated using ELISA and qRT-PCR. As shown in Fig 2, the addition of LPS had increased NO production remarkably. However, 10^7^ and 10^8^ CFU/mL of bacterial isolates considerably inhibited the production of NO in a concentration-dependent manner. Particularly, 4M13 and 4B15 showed the greatest inhibitory effect on NO production. Moreover, the two strains substantially inhibited the release of inflammatory mediators such as TNF-α, IL-6, IL-1β, and IL-10 stimulated by the LPS treatment in a concentration-dependent manner in the further experiments using ELISA and qRT-PCR (Fig 3).

### Study of the genome properties and comparative analysis of the selected *Lactobacillus* strains

**Fig 4:**
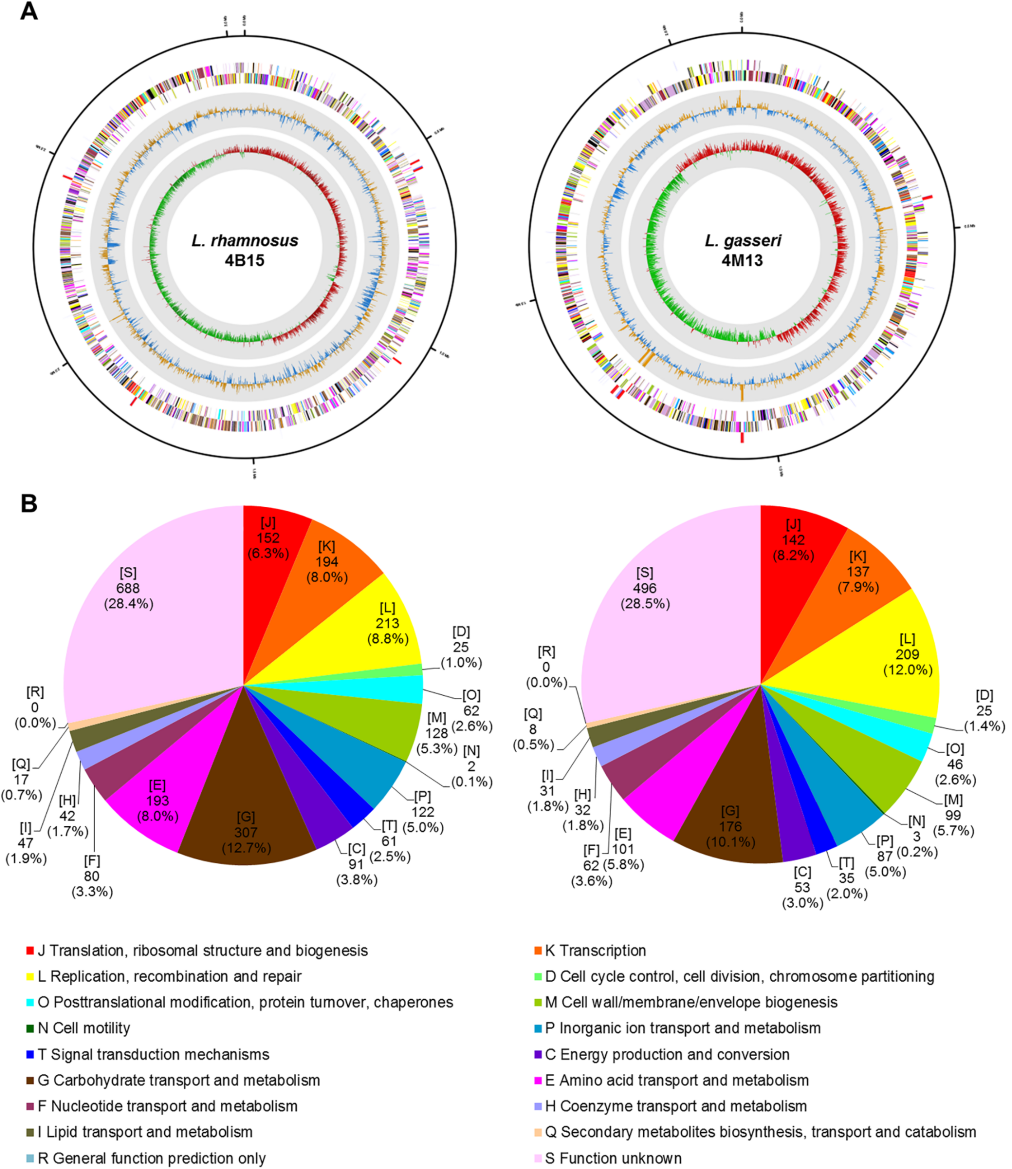
High-throughput genome sequencing of the selected *Lactobacillus* strains. A. Circular map of the selected *Lactobacillus* strains genome. Antisense and sense strands (colored according to COG categories) and RNA genes (red, tRNA; blue, rRNA) are shown from the outer periphery to the center. Inner circles show the GC skew, with yellow and blue indicating positive and negative values, respectively, and the GC content is indicated in red and green. This genome map was visualized using CLgenomics. B. Relative abundance of cluster of orthologous groups (COG) functional categories of genes.

To obtain functional information, whole genome sequencing and comparative genomic analysis of two selected strains, 4B15 and 4M13 were performed. The general genomic features of the strains are shown in Table 2, and circular genome maps of the strains are shown in Fig 4A. A total of 91,317 reads with an average length of 3,040,074 bp (G + C content of 46.68%) was obtained from 4B15, while the genome sequence of 4M13 was 2,114,362 bp (G + C content of 34.94%) in length with a total of 95,521 reads. The genome sequences of 4B15 and 4M13 consisted of two contigs for each strain with N50 values of 3,016,126 bp and 2,060,681 bp, respectively. Moreover, the genomes of 4B15 and 4M13 contained 2,863 and 2,089 coding DNA sequences (CDS), 15 and 12 rRNA genes, and 59 and 60 tRNA genes, respectively. The distribution of the COGs is illustrated in Fig 4B. The most abundant COG categories were G (carbohydrate transport and metabolism), L (replication, recombination, and repair), K (transcription), J (translation, ribosomal structure, and biogenesis), and E (amino acid transport and metabolism). The S category (unknown function) was also abundant. The subsystems of “Carbohydrates”, “Phages, Prophages, Transposable elements, Plasmids”, “Cell Wall and Capsule”, “DNA Metabolism”, “Membrane Transport”, “Clustering-based subsystem”, and “Amino Acids and Derivatives” based on the SEED database for both strains.

**Table 2:** General genomic information of the selected *Lactobacillu*s strains.

	*L*. *rhamnosus* 4B15	*L*. *gassei* 4M13
Sequencing platforms	Pacbio_20K	
Assembler	Pacbio SMRT Analysis 2.3.0	
methods reads	91,317	95,521
Methods coverage	421.71	573.42
Genome size (bp)	3,040,074	2,114,362
G+C content (%)	46.68	34.94
Predicted CDS	2,863	2,089
Number of contigs	2	2
Number of rRNA genes	15	12
Number of tRNA genes	59	60
N50 (bp)	3,016,126	2,060,681

**Fig 5:**
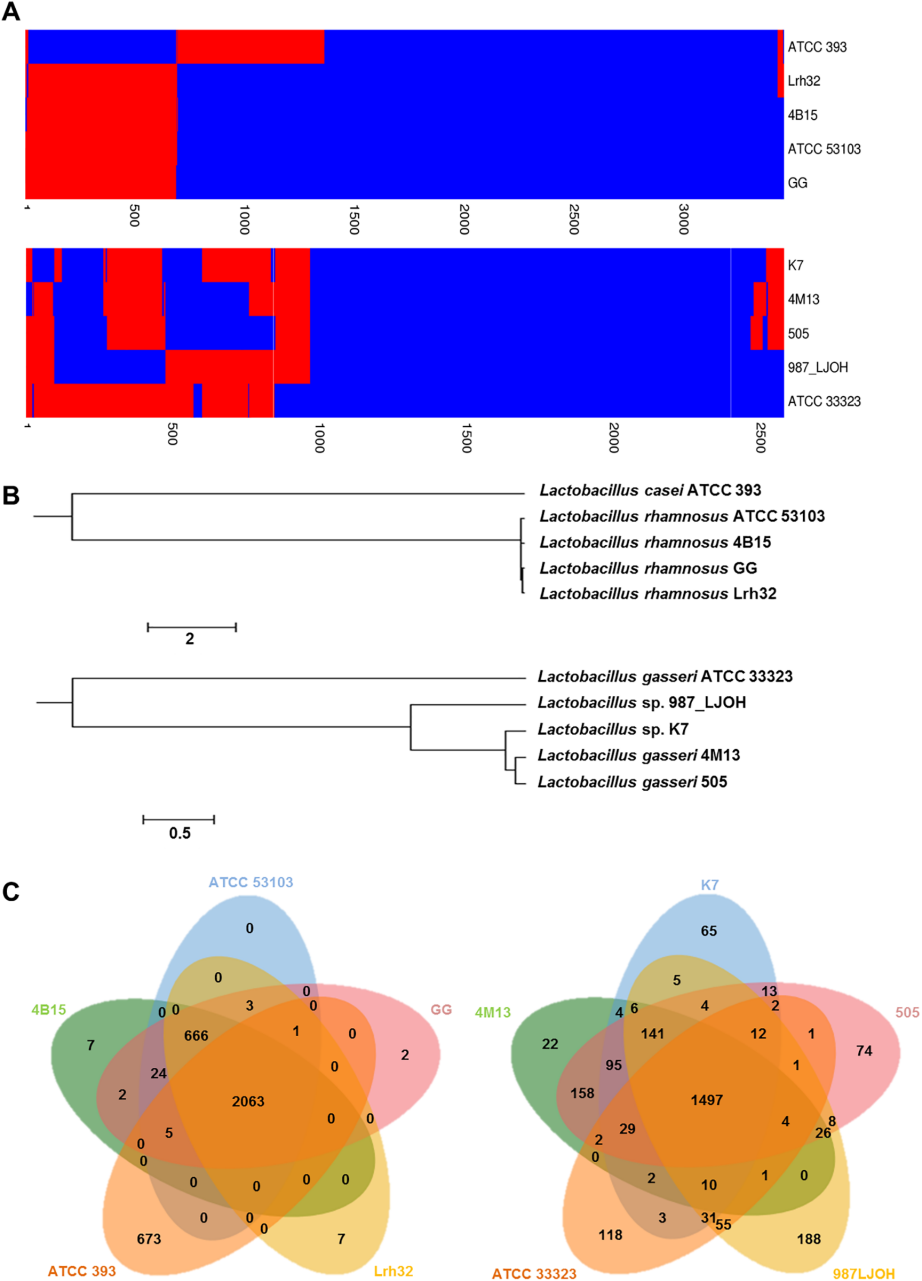
Comparative genomics of the selected *Lactobacillus* strains. A. Dendrogram based on presence of POGs. Using Jaccard coefficients and UPGMA clustering, a dendrogram was generated. Blue indicates present genes and red indicates absent genes. B. ANI phylogenetic tree. Using the orthologous average nucleotide identity tool, the phylogenetic tree was constructed based on OrthoANI values. C. Venn diagram representing the pan-genomic landscape of selected *Lactobacillus* strain and compared strains. The numbers in the Venn diagram indicate the number of POGs found to be shared among the indicated genomes.

Whole-genome comparison of the selected strains, 4B15 and 4M13 with four different reference strains is shown in Fig 5. The reference strains were highly homologous with 4B15 or 4M13 based on the analysis of gene presence or absence using a heat map, though the strains of 4B15 and 4M13 possessed POGs that were different from those of the reference strains (Fig 5A). In the dendrogram that was constructed based on the presence of POGs, the strain 4B15 was located close to the other strains. Moreover, the phylogenetic tree, which was constructed based on orthoANI values indicated that the two strains 4B15 and 4M13 were closely related to the reference strains (Fig 5B). The values of orthoANI of 4B15 and 4M13 with the reference strains exceeded 94% and 79%, respectively. As shown in Fig 5C, the strain 4B15 and other strains shared 2,063 POGs, while 4M13 shared 1,497 POGs with the reference strains. The genomes of 4B15 and 4M13 contained only 7 and 22 singletons, respectively. Only 2 POGs among 7 singletons in the genome of 4B15 were identified as part of the “Protein metabolism” and “Clustering-based subsystems” in the SEED database. The genome of 4M13 also consisted of 2 singleton POGs, which belonged to the “Clustering-based subsystem” and “Phage, prophage, transposable elements” in the SEED database. The four genes indicate the functions of protein biosynthesis, cell division, ATP-binding, and DNA integration, respectively.

## Discussion

Probiotic lactic acid bacterial strains should be able to survive the passage through the GI tract, preferably colonize the small intestine and colon for a sufficiently long period, and provide specific health benefits to the host [22]. The isolated *Lactobacillus* strains, especially 4B15 and 4M13 exhibited high tolerance to acid and bile and significantly higher adhesion activities to HT-29 cells. *Lactobacillus* species that are isolated from harsh environments are mainly acid-tolerant [23]. Previously, Jin et al. [24] and Guo et al. [25] reported that the *Lactobacillus* strains isolated from chicken caecum or koumiss showed only 60% ~ 85% survival after incubation for 3 h at pH 2–2.5, while the strains analyzed in this study had a survival rate of over 97.2%. Acid-tolerant strains are able to survive and resist the process of digestion in the stomach, where gastric juice and hydrochloric acid are secreted. Bile tolerance is considered an important characteristic of *Lactobacillus* strains, by which they grow and survive in the upper small intestine, since bile salts disorganize the structure of the cell membrane [3]. It has been shown that some probiotic strains overcome this problem through the production of bile salt hydrolase (BSH), which can break down conjugated bile salts, and decrease their toxicity [25]. The ability to adhere to intestinal epithelial cells is another important requirement for probiotic strains for their colonization, competition with pathogens and conferment of benefits to the host [26,27]. Moreover, the isolated *Lactobacillus* strains were observed to be urease- and gelatinase-negative in the assessment of safety. Based on the probiotic test, the eight strains, of which especially 4B15 and 4M13, exhibited the ability to survive the GI tract and colonize the human intestine, making potential probiotic candidates.

However, the functional properties of the isolates, such as anti-oxidation, inhibition of α-glucosidase activity, cholesterol-lowering, and anti-inflammation varied from strain to strain. Among the 8 strains, 4B15 and 4M13 exhibited the highest activities in the tests for all the functional properties. The highest antioxidant potential was observed in 4B15 and 4M13, particularly, 4B15, which showed a markedly high ORAC value, which indicated that both strains had a great ability to scavenge the peroxyl radical in both water- and lipid-soluble substances and high reducing power. Probiotic strains that have a high antioxidant potential may be useful in reducing oxidative damage and maintaining human health. Furthermore, 4B15 and 4M13 inhibited α-glucosidase activity, which could be attributed to the production of exopolysaccharides (EPS) by the *Lactobacillus* strains [28]. Lacroix and Li-Chan [29] reported that the inhibition of α-glucosidase activity is greatly affected by the origin of the enzyme because of structural differences in different enzymes. Mammalian (rat) intestinal α-glucosidase was used in this study, and thus, 4B15 and 4M13 could act as potential antidiabetic probiotics in the human body by reducing the intestinal absorption of carbohydrates. Both strains exhibited substantially higher cholesterol-lowering activity than the other tested strains. The cholesterol-lowering ability of the *Lactobacillus* strains could be due in part to the deconjugation of bile salts by BSH, which is produced by the strains [30]. The possible mechanism underlying the cholesterol-lowering ability of the *Lactobacillus* strains is the decrease of the solubility of cholesterol and thus, reduction of cholesterol uptake from the gut. NO is generated by phagocytes as part of the immune response. The inhibition of NO production in LPS-stimulated RAW 264.7 macrophages increased linearly with increasing concentrations of the *Lactobacillus* strains. In particular, 4B15 and 4M13 exhibited substantially higher inhibitory effects than other stains. Moreover, the levels of mRNA and proteins of TNF-α, IL-6, IL-1β, and IL-10 from RAW 264.7 had decreased proportionately with increasing concentration of 4B15 and 4M13, which could induce phagocytosis. Activated macrophages through phagocytosis regulate the immune system. These results suggest both strains exhibits elevated inhibition of the inflammatory cytokine expression in transcriptional level. Many reports have demonstrated the use of probiotics to ameliorate inflammation. *L*. *plantarum* 10hk2 has been shown to increase the production of pro-inflammatory mediators, such as IL-1β, IL-6, and TNF-α in RAW 264.7 cells [31]. Moreover another probiotics, *Bifidobacterium lactis*, has been shown to ameliorate experimental colitis as well as to exhibit *in vitro* anti-inflammatory activities [32]. In summary, the results of the analysis of the probiotic and functional properties of the *Lactobacillus* strains isolated from infant feces showed that the two strains, 4B15 and 4M13 in particular, are able to survive the GI tract and adhere to the intestinal mucosa to exert their beneficial effects such as anti-oxidation, inhibition of α-glucosidase activity, cholesterol-reducing and anti-inflammation, which were determined based on *in vitro* experiments. Therefore, the strains could be used as potential probiotics in the food and dairy industry.

In addition, whole genome sequencing of the two potential probiotic strains was performed, and comparative genome analysis of the isolated strains and four other publicly available *Lactobacillus* strains for each potential probiotic strain was conducted. Based on 16S rRNA phylogeny and genome sequence comparison including presence or absence of POGs and ANI values, 4B15 and 4M13, which were isolated from infant feces, were concluded to be novel genomic strains. However, the strain 4B15 shared a considerable number of POGs with *L*. *rhamnosus* GG (ATCC 53103). The two *L*. *rhamnosus* strains are some of the best characterized probiotic bacteria, and the genomes of the strains are well established [33,34]. There might be the evidence that the strain 4B15 provides important health benefits. Interestingly, the genome sequence of 4B15 contained 101 genes encoding putative proteases including oppA (LC4B15_01504), dtpT (LC4B15_02406), and dppC, dppE, and dppF, while oppA, oppF, and dtpT were also encoded in the 4M13 genome. These genes encode the peptidases involved in the processing of bioactive peptides. Moreover, the genomic analysis of both the 4B15 and 4M13 strains revealed the presence of oxidative stress response genes such as gshA, tpx, and ahpC and immune related genes (LC4B15_02082 through 02084 and LG4M13_01743 through 01745). Furthermore, strain-specific genes and gene clusters in 4B15 and 4M13 including those encoding various putative amino acid ABC transporters, glucosidases, and enzymes involved in bacteriocin synthesis were suspected to encode some of the unique features related to their probiotic and functional properties. In particular, the programmed ability to use fermentable proteins and sugars and produce beneficial substances involved in the antioxidant and anti-inflammatory properties of 4B15 was proved in a previous study by Oh et al. [35]. The genomic information of the novel *Lactobacillus* isolates 4B15 and 4M13 has enabled us to elucidate the genomic basis of their probiotic properties, and has provided us with fundamental knowledge for future applications in the food and dairy industry.

## Supporting information

S1 Table Identification of *Lactobacillus* strains using MALDI-TOF mass spectrometry. (DOCX)

S2 Table Safety test of *Lactobacillus* strains using urease and gelatinase tests. (DOCX)

S1 Fig Effect of *Lactobacillus* strains on RAW 264.7 macrophage cell viability. (TIF)
