# Supplementary material for: Probiotic and anti-inflammatory potential of Lactobacillus rhamnosus 4B15 and Lactobacillus gasseri 4M13 isolated from infant feces
Source: PLoS One. 2018 Feb 14;13(2):e0192021. doi: 10.1371/journal.pone.0192021 (PMC5812581; doi:10.1371/journal.pone.0192021)
Supplement: S1 Table — (DOCX) [file pone.0192021.s001.docx]

| **Strain** | **Species identification by MALDI-TOF/MS** | **No. of NCBI accesion** |
| --- | --- | --- |
| 3M02 | *Lactobacillus reuteri* | DSM 20016T |
| 3M03 | *Lactobacillus reuteri* | DSM 20056 |
| 4M13 | *Lactobacillus gasseri* | DSM 20604 |
| 4R22 | *Lactobacillus gasseri* | DSM 20077 |
| 5R01 | *Lactobacillus gasseri* | DSM 20604 |
| 5R02 | *Lactobacillus gasseri* | DSM 20604 |
| 5R13 | *Lactobacillus gasseri* | DSM 20604 |
| 4B15 | *Lactobacillus casei* | DSM 20011T |
