# Supplementary material for: Probiotic and anti-inflammatory potential of Lactobacillus rhamnosus 4B15 and Lactobacillus gasseri 4M13 isolated from infant feces
Source: PLoS One. 2018 Feb 14;13(2):e0192021. doi: 10.1371/journal.pone.0192021 (PMC5812581; doi:10.1371/journal.pone.0192021)
Supplement: S2 Table — (DOCX) [file pone.0192021.s002.docx]

| **Strain** | **Urease test** | **Gelatinase test** |
| --- | --- | --- |
| 3M02 | – | – |
| 3M03 | – | – |
| 4M13 | – | – |
| 4R22 | – | – |
| 5R01 | – | – |
| 5R02 | – | – |
| 5R13 | – | – |
| 4B15 | – | – |
