# Supplementary figures and images for: Probiotic and anti-inflammatory potential of Lactobacillus rhamnosus 4B15 and Lactobacillus gasseri 4M13 isolated from infant feces
Source: PLoS One. 2018 Feb 14;13(2):e0192021. doi: 10.1371/journal.pone.0192021 (PMC5812581; doi:10.1371/journal.pone.0192021)

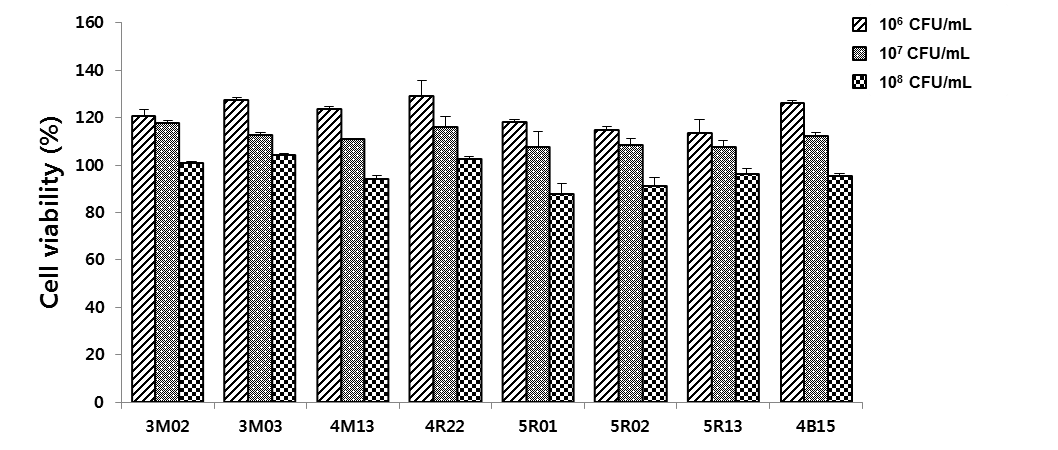

Supplement: S1 Fig — (TIF) [file pone.0192021.s003.tif]
